# 167 Acceptability and Feasibility of a Pharmacist-Driven Penicillin Allergy Delabeling Intervention in Patients with Hematologic Malignancy

**DOI:** 10.1017/ash.2026.10568

**Published:** 2026-06-23

**Authors:** Aryeh Feldheim, Kristyn Schumacher, Rahel Bahru, Frank Myers, Ian Jenkins, Robert El-Kareh, Francesca Torriani

**Affiliations:** 1 UC San Diego Health; 2 UCSD; 3 UC San Diego

## Abstract

**Background:** The clinical indication for vascular access devices (VAD), including central venous catheters (CVC) and peripheral intravenous catheters (PVC), should be assessed daily. If VADs are no longer indicated, they should be removed to reduce the risk of catheter-associated bloodstream infections (CABSI). In 2030, the CDC’s National Healthcare Safety Network (NHSN) will adopt a new metric for BSI surveillance called Hospital Onset Bacteremia (HOB) which is device agnostic. In this framework, all VADs, not just CVCs, will be considered potential causes of BSIs. We determined that unnecessary VAD use was increasing the hospital’s CLABSI rate, particularly in non-ICU units. We partnered with the Patient Safety Committee and the Mission Control (MC) center to develop an intervention, called MC-CABSI, to encourage prompt removal of unnecessary VADs. **Methods:** We developed five messages to communicate with patient care teams via our EHR’s secure chat, using data sourced from nursing flowsheets. The first, “Central Line Not Infusing,” tracks CVCs without documentation of infusion in the last 48 hours. The second and third are “Central Line and Two or More PVCs” and “Three or More PVCs,” respectively. These messages indicate high Vascular Access Device Density (VADD), defined as a unit’s median VAD days, including both CVCs and PVCs. The VADD accounts for multiple devices in a given patient, unlike the Standardized Utilization Ratio currently used for CLABSI reporting. The fourth message is “Central Line Not Meeting Necessity,” indicating a CVC documented as no longer indicated, but has not been removed. The fifth category is for any “Field-Inserted PVC,” present <24 hours after admission. Our policy requires such PVCs be removed due to the likelihood they were placed in sub-optimal conditions. Messages are sent by two infection preventionists daily and include one nurse and Attending. **Result:** We have sent 253 messages since the intervention began July 2025, and care teams responded by removing ≥1 CVC or PVC from 145 patients (57% response rate). Hospital-wide VADD has decreased from 1.40 to 1.33 over this time compared to the previous six months. **Conclusion:** Dialogue with care teams has been collaborative, with many expressing gratitude for this effort. As the intervention matures, we intend to track results, dialogue with providers, and hone messaging to improve outcomes. We see potential to expand the real-time feedback to cover other devices like indwelling urinary catheters. We also see opportunity for automation to improve scalability in preparation for the shift to HOB surveillance.

